# Activity Profile of an FDA-Approved Compound Library against *Schistosoma mansoni*

**DOI:** 10.1371/journal.pntd.0003962

**Published:** 2015-07-31

**Authors:** Gordana Panic, Mireille Vargas, Ivan Scandale, Jennifer Keiser

**Affiliations:** 1 Department of Medical Parasitology and Infection Biology, Swiss Tropical and Public Health Institute, Basel, Switzerland; 2 University of Basel, Basel, Switzerland; 3 Drugs for Neglected Diseases initiative (DNDi), Geneva, Switzerland; University of New Mexico, UNITED STATES

## Abstract

**Background:**

As plans to expand mass drug treatment campaigns to fight schistosomiasis form, worries about reliance on praziquantel as the sole available treatment motivate the investigation for novel antischistosomal compounds. Drug repurposing might be an inexpensive and effective source of novel antischistosomal leads.

**Methodology:**

1600 FDA approved compounds were first assayed against *Schistosoma mansoni* schistosomula at a concentration of 10 µM. Active compounds identified from this screen were advanced to the adult worm screen at 33.33 µM, followed by hit characterization. Leads with complementary pharmacokinetic and toxicity profiles were then selected for *in vivo* studies.

**Principal Findings:**

The *in vitro* screen identified 121 and 36 compounds active against the schistosomula and adult stage, respectively. Further, *in vitro* characterization and comparison with already available pharmacokinetic and toxicity data identified 11 *in vivo* candidates. Doramectin (10 mg/kg) and clofazimine (400 mg/kg) were found to be active *in vivo* with worm burden reductions of 60.1% and 82.7%, respectively.

**Conclusions/Significance:**

The work presented here expands the knowledge of antischistosomal properties of already approved compounds and underscores variations observed between target-based and phenotypic approaches and among laboratories. The two *in vivo*-active drugs identified in this study, doramectin and clofazimine are widely available and present as novel drug classes as starting points for further investigation.

## Author Summary

For a disease of large global health importance, schistosomiasis has a disproportionally small treatment tool box- only praziquantel is used to treat all 3 major forms of the disease. While drug discovery can be a long, laborious and expensive process, especially for an under-funded neglected disease such as schistosomiasis, drug recycling (also termed repositioning or repurposing) can bypass some of the development processes and offset the costs. We conducted a drug screening project of 1600 FDA-approved compounds from a very diverse set of indications against *Schistosoma mansoni*. The full 1600 compounds were first screened *in vitro* against the larval stage of the worm, of which 121 drugs were identified as active. These hits were then screened on the adult stages of the worm *in vitro* where 36 of these hits were also found to be active on the adult stage. The safety and pharmacokinetic profiles of hit compounds were then compared to their *in vitro* activity and 11 compounds were chosen for studies in mice. Of these, clofazimine and doramectin were found to be moderately active, and present new antischistosomal scaffolds with which further investigations can be pursued. Our findings are placed in context with results obtained from previous *in vitro* and *in silico* chemogenomics work and agreements and disagreements discussed.

## Introduction

Worldwide, schistosomiasis continues to affect the health and quality of life of millions, causing 3.3 million Disability-Adjusted Life-Years lost [1]. Most of the burden is contained in the tropics, mostly in Sub-Saharan Africa, where it disproportionally affects children in poor rural areas [2]. The three principal causative agents are *Schistosoma mansoni*, *Schistosoma haematobium* and *Schistosoma japonicum*. Infection with any of these three species, when left untreated, results in chronic inflammation which slowly develops into swelling, fibrosis and necrosis of the tissues of intestinal organs, the liver or the bladder, as well as a range of other symptoms which gradually impair the host physiologically and even cognitively [3,4].

The World Health Organisation (WHO) places morbidity control as a priority for treating schistosomiasis via preventative chemotherapy in the form of mass drug administration campaigns. Treatment targeted at high risk groups, mainly school-aged children, interrupts advancement to the cumulative damage of chronic stages, which causes most of the disease burden [5]. To date, this is seen as the most cost-effective strategy, as interruption of transmission is very difficult, costly and subject to many factors, and vaccine development is still far out of reach [6,7]. Yet of the 207 million people infected annually, in 2012 only 35 million received treatment at a given time [4]. Therefore, it has become essential to expand mass treatment campaigns. Indeed, as many as 235 million children are targeted to receive treatment by 2018 [8].

Nonetheless, we still rely on praziquantel as the sole treatment and the expanded use of this drug, while positively reducing morbidity, would also increase the potential for praziquantel resistance [9,10]. Regardless of expansion plans, reliance on one single drug for mass treating a population is dangerous. The international community has repeatedly stated the need for new medication, since the drug discovery and development pipeline is dry [11]. Earlier, we reviewed the ways in which drug repurposing is aiding helminth drug discovery [12] and highlighted several clinical success stories such as antimalarials for the treatment of schistosomiasis. Drug repurposing (or repositioning) is the development of new indications from existing, failed or abandoned drugs and offers some obvious benefits: researchers can piggy-back off the availability of pre-clinical data, saving time and costs, making more informed decisions on hit-to-lead identification and ultimately decreasing the time it takes to bring a drug to market [13,14].

In the framework of a Gates-funded drug discovery project, overseen by the Drugs for Neglected Diseases initiative (DND*i*), different libraries, including a library of 1600 FDA approved compounds with diverse classes and initial applications, were screened on *Schistosoma mansoni*. Abdulla and colleagues had previously evaluated a similar library of 2160 compounds on a Puerto Rican strain of *S*. *mansoni*, finding many *in vitro-*active compounds but no strong *in vivo-*active candidates [15]. Considering our past experience with strain and hit cut-off differences [16], we were encouraged to screen the above-mentioned 1600 compound FDA library in the hopes of identifying strong candidates to test *in vivo* and to compare our findings.

In more detail, the full 1600 compound library was initially assayed on newly transformed schistosomula (NTS- the larval stage). Compounds that reduced NTS viability by 75% were further tested on adult worms and their activity was compared to their existing pharmacokinetic and toxicity profiles before initiating studies in a mouse-*S*. *mansoni* infection model. Finally, we compare our results with findings reported from the above-mentioned screen by Abdulla et al. and a recent target-based chemogenomics screen of a dataset of 2,114 proteins by Neves et al. [17], and discuss overlaps and contradictions.

## Materials and Methods

### Compounds and Media

The FDA Pharmakon compound library was purchased from MicroSource Discovery Systems, Inc. (USA). Compounds were delivered in microplates (10 mM, dissolved in DMSO) and kept at -80°C until use. For *in vivo* studies, flunarizine hydrochloride, pimozide, nicardipine hydrochloride, oxethazaine, menadione, clofazimine, doramectin and metitepine mesylate were purchased from Sigma-Aldrich (Buchs, Switzerland) and fendiline hydrochloride, manidipine hydrochloride and lomerizine hydrochloride were purchased from Santa-Cruz Biotechnology (California, USA). Hanks Balanced Salt Solution (HBSS) was obtained from Gibco (Lucerne, Switzerland). Culture medium components for NTS and adult worms were obtained as follows: Medium 199 RPMI 1640 and penicillin (100 U/ml) and streptomycin (100 μg/ml) were purchased from Lubioscience (Lucerne, Switzerland) whereas inactivated fetal calf serum (iFCS) was purchased from Connectorate AG (Dietikon, Switzerland).

### *Schistosoma mansoni* Larval *In Vitro* Assay

NTS were obtained using a transformation method described previously [18]. Briefly, cercariae (Liberian strain) were harvested from infected intermediate host snails (*Biomphalaria glabrata*) after several hours’ exposure to light. The collected cercarial suspension was cooled, centrifuged and pipetted, and vortexed vigorously in HBSS to remove the tails. The suspension was rinsed in cool HBSS to remove the tails and the resulting NTS suspension was adjusted to a concentration of 100 NTS per 50 μl in NTS culture medium (Medium 199 supplemented with 5% iFCS and 1% penicillin/streptomycin). The NTS suspension was then incubated at 37°C, 5% CO_2_ in ambient air for 24 hours. Drugs were first tested at a concentration of 10 μM on NTS. The worms were incubated in culture medium and the test compounds in a 96-well plate in triplicate for 72 hours. Thereafter, they were assessed microscopically using a viability scale previously described [16], which scores the morphology and motility of the NTS (3 = motile, no changes to morphology; 2 = reduced motility and/or some damage to tegument noted; 1 = severe reduction to motility and/or damage to tegument observed; 0 = dead). Hits were characterized as compounds that achieved an average viability score of 0.5 or less (corresponds to NTS viability of ≤ 25%).

### *In Vitro* Drug Sensitivity Assay on *Schistosoma mansoni* Adult Worms

Compounds identified as hits in the NTS drug assay were further tested on adult worms. Mice were infected as detailed in the *in vivo* studies section below and the infection was allowed to mature for 7 weeks. Mice were then euthanized with CO_2_ and their intestinal apparatus was dissected. Worms were collected from the hepatic portal and mesenteric veins and subsequently rinsed and stored in culture medium (RPMI supplemented with 5% iFCS and 1% penicillin/streptomycin) at 37°C, 5% CO_2_ until use. In a 24-well plate, 2–4 worm pairs were placed in culture medium and 33.33 μM of the test compound for 72 hours, 2 wells per compound. Effects were assessed microscopically with the same viability scale used for NTS and again, compounds that achieved an average score of 0.5 or less but after 24 hours were considered as hits. For further hit characterization, the IC_50_ values were determined in an adult worm dose-response assay (33.33, 11.11, 3.70, 1.43 and 0.41 μM drug concentration) at 1, 2, 4, 7, 10, 24, 48 and 72 hours post-drug incubation.

### *In Vivo* Studies in the *S*. *mansoni* Mouse Model

For *in vivo* studies, female 3-week old NMRI mice were used. Mice were purchased from Charles River (Sulzfeld, Germany) and allowed to adapt under controlled conditions (temperature ca. 22°C; humidity ca. 50%; 12-hour light and dark cycle; free access to rodent diet and water) for one week. Thereafter, they were infected subcutaneously with approximately 100 *S*. *mansoni* cercariae (obtained as described above). Seven weeks post-infection, 4 mice were assigned to each drug treatment, while 8 mice were left untreated to serve as controls. Compounds were prepared in a 70:30 Tween/EtOH mixture dissolved in dH_2_O (10%). Available compound toxicity data was used to guide the dosing regimen. Compound doses were adjusted to the mouse weight and were administered orally. Three weeks post-treatment, mice were killed by the CO_2_ method and dissected, and the worms were sexed and counted. Mean worm burdens of treated mice were compared to the mean worm burden of untreated animals and worm burden reductions were calculated.

### Ethics Statement

*In vivo* studies were conducted at the Swiss TPH, Basel, and approved by the veterinary authorities of the Canton Basel-Stadt (permit no. 2070) based on Swiss cantonal (Verordnung Veterinäramt Basel-Stadt) and national regulations (the Swiss animal protection law (Tierschutzgesetz).

### Statistics

For *in vitro* assays, the viability scores were averaged across replicates and normalized to control-well viability scores using Microsoft Office Excel (2010). IC_50_ values were computed using CompuSyn2 (ComboSyn Inc., 2007) by converting viability scores into effect scores for each drug concentration.

The worm burden (WB) of treated mice was calculated and compared with the worm burden of control mice in order to obtain the worm burden reduction (WBR), calculated as follows:

$$\text{WBR}\;\left(\%\right)\;=\;100\%\;-\;\left(100\%\;/\;{\text{WB}}_{\text{control}}\times \;{\text{WB}}_{\text{treatment}}\right)$$

Statistical comparison was done using the Kruskal Wallis Test and the Mann Whitney U test at a significance level of p < 0.05.

## Results

### *In Vitro* Activity on NTS and Adult *S*. *mansoni*

**Fig 1:**
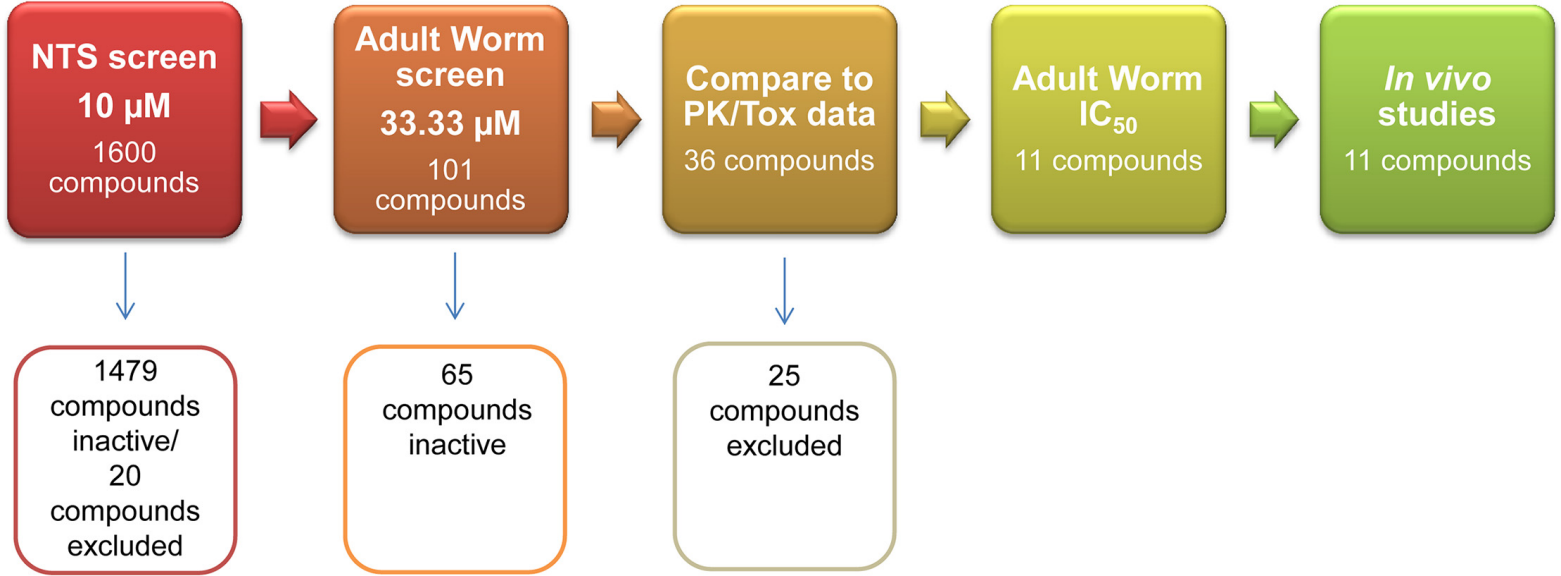
*In vitro* screening cascade of the FDA Pharmakon library against *S*. *mansoni* NTS and adult worms.

The overall screening cascade is presented in Fig 1.

Of the 1600 compounds screened on NTS, 121 compounds (summarized by indication in Table 1) showed activity at a concentration of 10 μM after 72 hours. Of these, 57 compounds killed the NTS completely within 72 hours of exposure and 64 compounds damaged the NTS severely (viability score ≤ 0.5, corresponding to a viability of ≤ 25%) within the same time frame. After a quick scan of the hits, 20 compounds were excluded due to their known high toxicity (e.g. colchicine) or because their activity against *S*. *mansoni* has already been described (e.g. mefloquine) (Table 1).

**Table 1:** 121 Hit compounds at NTS screening stage.

**Antibacterial**	Cefdinir*; Chloroxine; Clofoctol*; Gentian violet; Hexachlorophene; Lasalocid sodium; Monensin sodium*; Narasin; Natamycin; Pyrithione zinc; Salinomycin sodium*; Sulfanitran; Thonzonium bromide;	**Antipsychotic**	Aripiprazole; Chlorprothixene hydrochloride*; Fluphenazine hydrochloride; Metitepine mesylate; Perphenazine; Pimozide; Prochlorperazine edisylate; Sertraline hydrochloride (anti-depressant); Thioridazine hydrochloride; Trifluoperazine hydrochloride; Triflupromazine hydrochloride*;
**Antifungal**	Broxaldine; Butoconazole; Candicidin; Ciclopirox olamine; Econazole nitrate; Hexetidine; Itraconazole hydrochloride; Miconazole nitrate; Oxyquinoline sulfate; Phenylmercuric acetate; Piroctone olamine; Sulbentine; Sulconazole nitrate; Terbinafine hydrochloride; Thiram;	**Antihistamine/ anti-inflammatory**	Azelastine hydrochloride; Betamethasone sodium phosphate (also immunosuppressant); Cepharanthine; Cinnarazine; Escin; Montelukast sodium; Promethazine hydrochloride*; Terfenadine;
**Antiprotozoal**	Mefloquine**; Nifursol; Primaquine phosphate; Pyronaridine tetraphosphate;	**Statins/ Anti-diabetic/ coronary disease**	Fluvastatin; Lovastatin; Metformin hydrochloride (Diabetic); Orlistat; Perhexiline maleate
**Anthelminthic**	Abamectin; Antimony potassium tartrate trihydrate**; Doramectin; Eprinomectin; Hycanthone**; Moxidectin; Niclosamide; Oltipraz**; Praziquantel**; Pyrvinium pamoate; Quinacrine hydrochloride; Selamectin; Trichlorfon**	**Antihypertensive/ vasodilator**	Amlodipine besylate; Fendiline hydrochloride; Flunarizine hydrochloride; Lomerizine hydrochloride; Manidipine hydrochloride; Nicardipine hydrochloride; Prazosin hydrochloride*; Reserpine; Suloctidil; Vinpocetine
**Antiinfective/ antiseptic**	Acriflavinium hydrochloride; Benzalkonium chloride; Benzoxiquine; Bronopol; Cetrimonium bromide; Mepartricin; Methylbenzethonium chloride; Nifuroxazide; Thimerosal	**Cholinergic/ Anasthetic/ Cardiac stimmulant**	Atracurium besylate; Inamrinone; Neostigmine bromide*; Oxethazaine; Physostigmine salicylate*; Proscillaridin; Pyridostigmine bromide
**Anticancer**	Arsenic trioxide diethanolamine salt; Tamoxifen citrate; Toremiphene citrate;	**Other**	Acamprosate calcium; Amsacrine; Chlormadinone acetate; Clofazimine; Clomiphene citrate**; Colchicine*; Cyclosporine**; Digitoxin*; Erythrosine sodium; Idebenone; Iodipamide; Menadione; Methylene blue; Mifepristone; Octisalate; Podofilox; Protoporphyrin ix; Riboflavin; Riboflavin 5-phosphate sodium; Securinine; Tenylidone; Tolonium chloride; Tolperisone hydrochloride

Compounds were tested at a concentration of 10 μM and hits were defined as compounds for which the NTS scored ≤ 0.5 on our viability scale at 72 hours post-exposure.

*Indicates compound excluded due to toxicity concerns.

**Indicates compound that has already been well-characterized on *Schistosoma spp*. and hence was excluded.

From the NTS screen, therefore 101 compounds qualified for testing on *S*. *mansoni* adult worms, at a single high concentration of 33.33 μM. Of these, 36 compounds were found to be active 24 hours post-incubation: the compounds induced death of the worms or a 75% reduction in their viability (a final viability score of ≤ 0.5). However, of the 36 active compounds, 25 were excluded following a closer review for the following reasons: 8 were excluded due to known toxicity in humans, 9 were indicated for topical use only, 5 had been described to have toxicity in rodents (low LD_50_ values), 2 were excluded due to past or current studies conducted on *S*. *mansoni in vivo* models (niclosamide studied by Abdulla et al. (15); tamoxifen studied by Cowan et al, submitted for publication), and 1 was rejected due to its poor absorption (Table 2).

**Table 2:** Compounds active on adult *S*. *mansoni* at a concentration of 33.33 μM at 24 hours.

Indication	Compound	Exclusion
**Antibacterial**	Pyrithione zinc	Topical
	Gentian violet	Topical
	Hexachlorophene	Topical
	Thonzonium bromide	Topical
	Narasin	Toxicity in rodent
**Antifungal**	Miconazole nitrate	Topical
	Hexetidine	Topical
	Phenylmercuric acetate	Toxic
**Anthelminthic**	Doramectin	
	Abamectin	Toxicity in rodent
	Eprinomectin	Toxicity in rodent
	Pyrvinium pamoate	Poor absorption
	Niclosamide	Being studied
	Selamectin	Topical
**Anti-infective/antiseptic**	Methylbenzethonium chloride**	Topical
	Cetrimonium bromide	Topical
	Thimerosal	Toxic
**Anticancer**	Arsenic trioxide diethanolamine salt	Toxic
	Tamoxifen citrate	Being studied
**Antipsychotic**	Pimozide	
	Metitepine mesylate	
**Antihistamine/ anti-inflammatory**	Manidipine hydrochloride	
	Terfenadine	Toxic
**Statins/ Anti-diabetic/ coronary disease**	Perhexiline maleate	Toxic
**Antihypertensive/ vasodilator**	Fendiline hydrochloride	
	Flunarizine hydrochloride	
	Lomerizine hydrochloride	
	Nicardipine hydrochloride	
	Suloctidil	Hepatotoxic
	Amlodipine besylate	Toxicity in rodent
**Cholinergic/ Anesthetic/ Cardiac stimulant**	Proscillaridin	Toxicity in rodent
	Oxethazaine	
**Other**	Menadione	
	Clofazamine	
	Tenylidone	Topical
	Securinine	Toxic

Activity was defined as scoring an average of ≤ 0.5 on the viability scale. The reason for exclusion is also listed and the data is based on compound material safety data sheets, FDA documents and previous publications.

**Fig 2:**
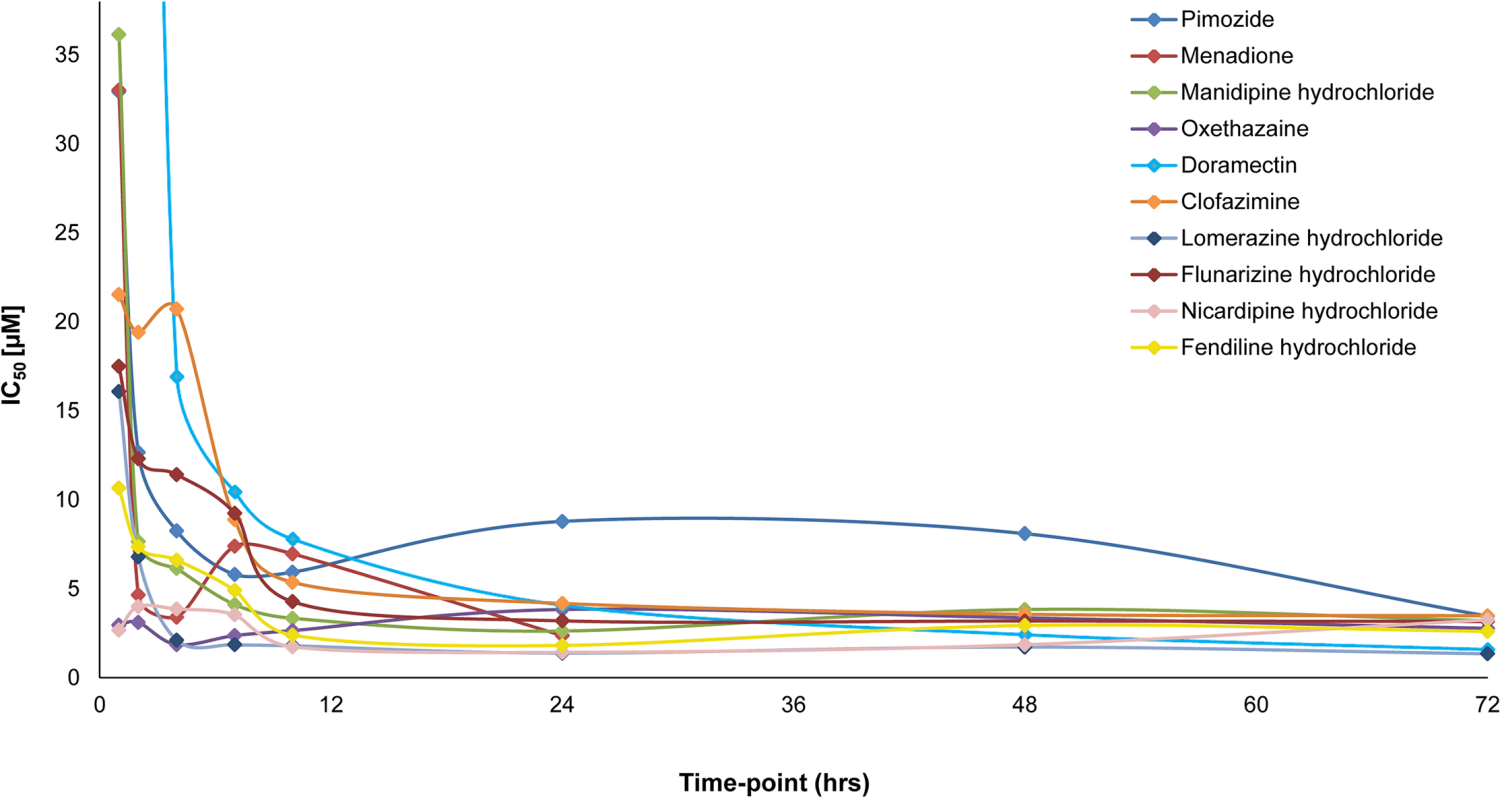
IC_50_ values of compounds selected for *in vivo* testing.

The remaining 11 compounds were further characterized with an IC_50_ determination assay at various time-points (Fig 2 and S1 Table). Already after 2 hours incubation, most compounds (except pimozide, doramectin, clofazimine and flunarazine hydrochloride) exhibited IC_50_ values below 10 μM, and by the 10 hour time-point, IC_50_ values for these compounds ranged from 1.73–7.80 μM. The fastest acting compounds were nicardipine hydrochloride and oxethazaine, exhibiting IC_50_ values of 2.67 and 2.95 μM respectively already at 1 hour post-incubation. Meanwhile, doramectin and clofazimine were the slowest acting, with IC_50_ values of 16.92 and 20.72 μM respectively at 4 hours post-exposure. Between 24 and 72 hours post-exposure, IC_50_ values did not vary greatly between drugs, ranging between 1.34 to 4.17 μM, except for pimozide which jumped to 8.78 μM at 24 hours and declined to 3.46 μM by the 72-hour time-point.

These timed IC_50_ values were compared to available pharmacokinetic data, and all 11 compounds were selected as good *in vivo* candidates. As previously mentioned, the *in vitro* data and available pharmacokinetic and rodent toxicity data were also used to guide the maximum possible single oral dose regimens.

### *In Vivo* Studies in the *S*. *mansoni* Mouse Model

Dosing, worm burden and worm burden reductions of the 11 compounds tested are presented in Table 3.

**Table 3:** Worm burden reductions of *S*.*mansoni-*infected mice treated with *in vitro-*hit FDA Pharmakon compounds.

			Worm Burden		Worm Burden Reduction (%)	
Compound	Dose (mg/kg)	No. of mice	Female	Total	Female	Total
Control Batch 1	Untreated	8	24.1	50.1	-	-
Control Batch 2**	Untreated	8	14.8	31.3	-	-
Clofazimine	400	3*	3.7	8.7	80.8	82.7
Clofazimine**	200	4	19.8	38.5	0	0
Doramectin	10	4	10.3	20.0	62.5	60.1
Fendeline hydrochloride	100	3*	34.0	68.7	0	0
Flunarizine hydrochloride	200	4	18.0	36.8	27.9	26.7
Lomerizine hydrochloride	200	4	24.8	52.5	0	0
Manidipine hydrochloride	100	4	19.3	36.3	34.6	27.7
Menadione	400	4	27.3	53.3	0.0	0
Metitepine mesylate	All doses toxic- study stopped	N/A	N/A	N/A	N/A	N/A
Nicardipine hydrochloride	200	3*	12.3	25.3	50.0	49.5
Oxethazaine	200	3*	16.3	32.3	38.5	35.5
Pimozide	100	4	13.0	25.3	52.6	49.5

*Indicates that the 4^th^ mouse of this group died prematurely.

** WBR for clofazimine (200 mg/kg) was calculated based on worm burden of Control Batch 2.

Metitepine mesylate proved to be toxic to mice at each dose tested (400, 200, 100 and 50 mg/kg, one mouse tested per dose and observed) and further investigation with this compound was ceased. Doramectin exerted a moderate worm burden reduction (60.1%) and clofazimine caused a high, however not statistically significant worm burden reduction (82.7%). In a follow-up *in vivo* study, a dose 200 mg/kg clofazimine was tested in 4 *S*. *mansoni-*infected NMRI mice. Lowering the dose, however, resulted in a complete lack of efficacy (0% female and total WBR). Pimozide and nicardipine hydrochloride also demonstrated some efficacy (49.5% WBR for both), whereas mild WBRs were observed for flunarizine hydrochloride (26.7% WBR), oxethazaine (35.5% WBR) and manidipine hydrochloride (27.7% WBR). Lomerizine hydrochloride, fendeline hydrochloride and menadione lacked *in vivo* activity.

## Discussion

The advent of praziquantel in the 1970s was a great milestone for the control of schistosomiasis in that finally, a safe, cheap and effective drug became available that could be used to treat millions in cost-effective preventive chemotherapy campaigns. Unfortunately, the success of praziquantel also resulted in many labs and firms choosing to drop further investigations on their leads [19]. This, along with inadequate attention and funding has rendered the antischistosomal arsenal dangerously dependent on a single drug [10].

**Fig 3:**
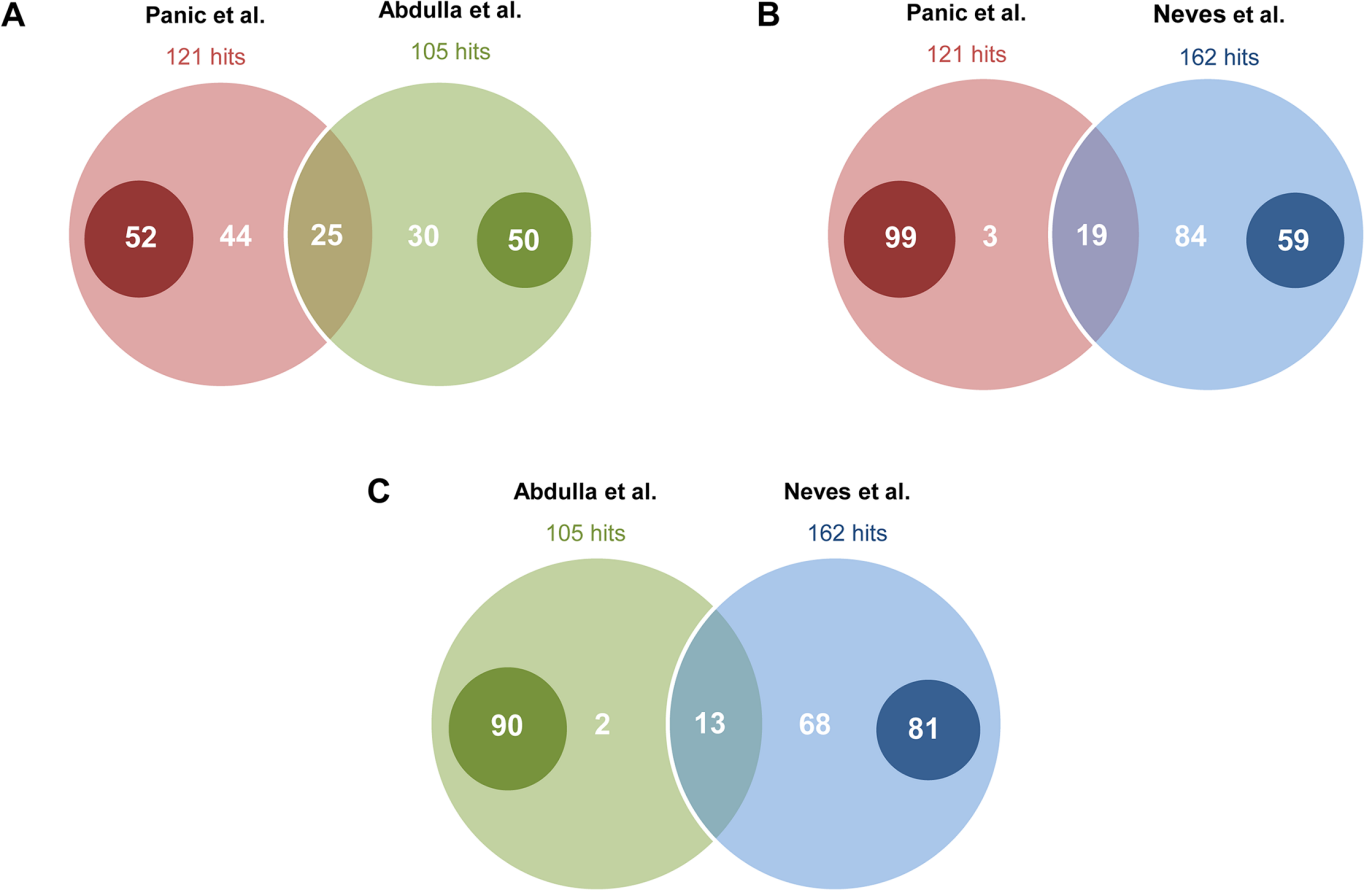
Venn diagrams representing overlaps between: (A) our NTS screen and that of Abdulla et al., (B) our NTS screen and *in silico* hits from Neves et al. and (C) NTS hits from Abdulla et al. and hits from Neves et al. The small dark circles within each large circle represent the number of compounds that were not present in the comparator’s library.

As *de novo* drug discovery becomes increasingly expensive, drug repurposing, on the other hand, has shown to bear fruit in the antischistosomal drug discovery field as well as others with fewer resources involved [12,20,21]. By screening a library of well-characterized compounds, it was our hope to identify new drugs or drug classes that could be explored further in pre-clinical development. An initial screen against NTS revealed a hit rate of ~7.6% and encompassed a wide range of compound indications including antipsychotics, antibiotics, antifungals, antihistamines, antihypertensives and even vitamin precursors and metabolites (Table 1). Results of this work in part mirrored the screen conducted by Abdulla and colleagues (mentioned earlier), in that the variety of active compounds also ranged across a large spectrum of indications [15]. However, in comparing our NTS hits, it was interesting to note that although there was some overlap, there were numerous incongruences as well (S2 Table and Fig 3A). Of the 121 NTS hits we identified in our library, 69 of the compounds were also found in their library, but of these 69, only 25 were identified as hits (36% overlap). Conversely, of the 105 hits identified in the library of Abdulla and colleagues, 55 were found in our library and of those, 25 were characterized as hits (45%). These inconsistencies are likely a combination of differences in drug concentrations used, time of evaluation post-drug exposure and screen cut-off filters for hit identification, which indicates that these factors can greatly influence the outcome of a screen. Indeed, a closer inspection of hits identified by Abdulla et al. and missed in our library revealed that most of these “missed compounds” had some effect on our NTS but not enough to reach the cut-off threshold. Nonetheless, it may also be possible that strain differences result in differing drug susceptibilities (Abdulla et al. used a Puerto Rican strain, whereas ours was a Liberian strain). Indeed, Ingram-Sieber et al. observed similar differential sensitivities from their screen of MMV Malaria Box compounds conducted by two independent labs using exactly these two strains [16]. Bearing these incongruences in mind, it might be useful to start a discussion on a possible need for standardization and replication.

Recently, Neves et al, took advantage of the published genome and transcriptome of *S*. *mansoni* [22] as well as public drug databases to conduct an *in silico* screen of compounds with known targets that theoretically match targets found in the *S*. *mansoni* genome and transcriptome [17]. When we compared their hits to our NTS hits, a moderate overlap was observed (S2 Table and Fig 3B). In more detail, of the 162 compounds described to match to an *S*. *mansoni* target *in silico* (115 compounds they describe as novel along 47 with compounds for which some activity has already been described), 102 were present in our library, and of these 102, 19 were deemed as active, corresponding to a 19% overlap. The fact that the *in silico* prediction did not strongly match our *in vitro* hits, doesn’t necessarily mean the *in silico* hits are incorrect: they could be differentially active on other life stages (ex. juvenile), active *in vivo*, at a higher concentration, or, as we saw with Abdulla and colleagues, in a different screen with a different strain. Nonetheless, it does hint that target-based approaches still require further development.

**Fig 4:**
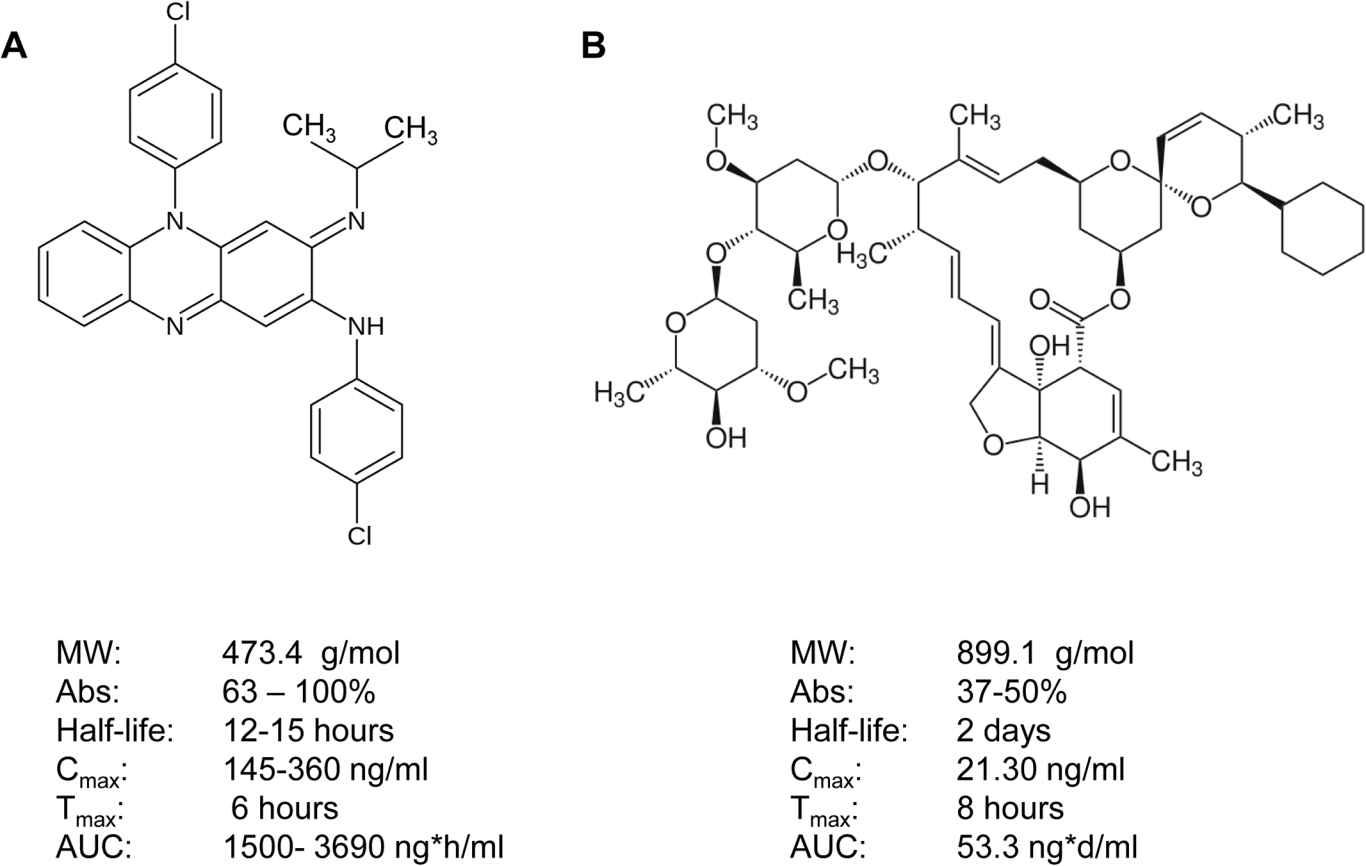
Structures and pharmacokinetic data of *in vivo-*active clofazimine (A) and doramectin (B). Clofazimine data is based on a single oral dose of 200 mg give to healthy male volunteers [45]. Doramectin data is based on a single oral dose of 200 μg/kg administered to horses [46].

In this screen we identified doramectin and clofazimine as two moderately active compounds against *S*. *mansoni* in an NMRI mouse infection model (Fig 4).

The activity of doramectin, though not statistically significant, is nonetheless surprising: studies in *S*. *mansoni-*infected mice with the highly related ivermectin showed minimal efficacy when administered as a single oral dose of 25 mg/kg [23]. Moreover, clinical trials with ivermectin showed little efficacy against intestinal and urinary schistosomiasis [24]. However, doramectin is reported to have more favorable pharmacokinetic properties, which could account for its higher *in vivo* efficacy in our study [25]. Doramectin has not been previously studied in humans and therefore would need substantial efforts to register the drug for human medicine. However, the closely related moxidectin has shown a moderate effect against *S*. *mansoni* in preliminary clinical studies [26]. Moxidectin was active against NTS and moderately active against the adult stage worm in our screen, and hence may be worthy of further investigation. Clofazimine is originally indicated for treatment against leprosy and is on the WHO Model List of Essential Medicines [27]. It is a fat-soluble iminophenazine dye that has demonstrated immunosuppressive properties, including inhibition of macrophages, neutrophil motility, lymphocyte transformation and mitogen-induced PBMC formation [28–30]. Recently, clofazimine was also identified as a promising preclinical anti-trypanosomal agent in an *in silico* screen of marketed drugs [31]. The authors noted that the compound was also effective in inhibiting epimastigote proliferation *in vitro* and in reducing parasitaemia levels in a murine infection model at a dose of 20 mg/kg. In our study, the initial dose of 400 mg/kg clofazimine showed a moderately high WBR (82.7%), while lowering the dose to 200 mg/kg was not effective. Considering the long half-life of clofazimine (12–15 hours) as well as its reportedly good absorption (60–100%), we deemed it unnecessary to study the effects of multiple dosing. Currently, off-label use of clofazimine is highly discouraged by the WHO, as it is the first line of treatment against leprosy and there are legitimate fears of drug resistance [32]. Nonetheless, it may be worth exploring the antischistosomal activities of related structures.

With the advent of the *S*. *mansoni* genome, there have been attempts to incorporate rational drug screening in the antischistosomal drug discovery process. Recently, the observation that fatty acids play a major role in schistosome development, fecundity and tegument construction has spurred researches to investigate the potentials of cholesterol-lowering statins [33–36]. Consequently, Rojo-Arreola and colleagues evaluated six statin compounds, atorvastatin, fluvastatin, lovastatin, pravastatin, rosuvastatin and simvastatin, all targeting 3-hydroxy-3-methylglutaryl coenzyme A reductase (HMGR) of the eukaryotic mevalonate pathway on *S*. *mansoni* NTS and adult worms. All drugs were found to be active *in vitro* and targeted SmHMGR [37]. In our own screen, fluvastatin and lovastatin were found to be active on NTS but inactive on adult worms. Atorvastatin and simvastatin were inactive already at the NTS stage and pravastatin and rosuvastatin were not present in our library. The contrasting results could be partly attributed to the stricter cut-off parameters used in our screen (shorter drug incubation time, only severe reduction in viability considered) or could also be due to strain differences (the use a Puerto Rican strain). *In vivo* tests would be required to say anything substantial about the potential of statins as antischistosomals.

Considering rational *S*. *mansoni* drug targets, it was interesting to note that none of the calcium channel blockers such as fendeline hydrochloride or flunarizine hydrochloride showed a potent *in vivo* effect. In light of the notion that praziquantel’s mode of action is highly suspected to be due its disruption of Ca2+ homeostasis, the idea that known calcium channel blockers could be effective against *S*. *mansoni in vivo* is not too far-fetched [38,39]. That being said, these drugs are often used to treat chronic human disorders such as hypertension, migraines or allergies, meaning they also bind to human receptors. The fact that these drugs were very potent *in vitro* but not *in vivo* could be attributable to many factors such as drug metabolism or protein binding, but competition between host and parasite receptors might play a role. This could be a general drawback to repurposing drugs with known human receptor targets for use against parasite infections.

Indeed, while drug repurposing can potentially reduce the time and costs of the drug discovery process, its limitations should also be carefully considered and are already observable in our study. Although compound libraries intended for new indication screens often contain already marketed drugs, their safety window may not necessarily be acceptable for schistosomiasis treatment and preventative chemotherapy. Drug repurposing is a popular strategy for an array of diseases, some of which a narrow safety window is acceptable due to the nature of the disease [40,41]. Compounds chosen for development against schistosomiasis, however, must have an excellent safety profile, as they will be very widely used in preventive chemotherapy campaigns mainly targeted towards children [42]. Indeed, of the 36 adult worm hits, it was disappointing to note that 25 of these compounds were unsuitable for testing, notably due to documented severe side-effects, restriction to topical use or low LD_50_ values in mice. Some compounds, for example terfenadine, had even been withdrawn from FDA approval or were no longer marketed [43]. It would be favorable if further libraries of already known compounds would be strictly composed of drugs currently on the market with a good safety profile, in order for a real drug repurposing effort to be possible.

A further major challenge to drug repurposing might be the difficulty to develop a dose regimen in humans that provides plasma exposure in the range of the *in vitro* IC_50_ concentration, which tends to be high for helminths. Hence the chance that safety, pharmacokinetics and pharmacological action will match for a very different indication is uncertain. With these limitations in mind, the NTD community should not rely on drug repurposing alone as a drug discovery strategy. Nonetheless, it is a very worthy venture: as previously stated, many of the anthelmintics used today were repurposed from veterinary applications [12]. Moreover, 90% of the drugs available today have secondary indications, showing that repurposing continues to be a popular strategy both for academia and industry [44]. Importantly, each marketed drug very likely has a library of analogues behind it, with which structure activity relationship for a hit expansion program can certainly be envisaged. Access to these analogues would facilitate an optimization program aiming at the identification of preclinical candidates.

In conclusion, by screening a library of 1600 well characterized compounds, we have identified dozens of compounds active against *S*. *mansoni in vitro*. Many of the compounds safety or pharmacokinetic profiles rendered them unfavorable for further exploration. Nonetheless, of the 11 compounds screened *in vivo*, we identified two compounds with moderate to high activities with which further investigations may result in novel compound class treatments.

## Supporting Information

S1 Table Table of IC_50_ values over exposure time of compounds selected for *in vivo* testing (complement to Fig 2). (DOCX)

S2 Table Comparison of NTS hits between our data (Panic et al.), that of Abdulla et al. (2009) and general *in silico* hits from Neves et al. (2015). Green indicates a hit, red a non-hit and grey means it was lacking from the library.
(DOCX)
