# Supplementary material for: Activity Profile of an FDA-Approved Compound Library against Schistosoma mansoni
Source: PLoS Negl Trop Dis. 2015 Jul 31;9(7):e0003962. doi: 10.1371/journal.pntd.0003962 (PMC4521867; doi:10.1371/journal.pntd.0003962)
Supplement: S1 Table — (DOCX) [file pntd.0003962.s001.docx]

|  | **IC_50_ (µM)** | | | | | | | |
| --- | --- | --- | --- | --- | --- | --- | --- | --- |
| **Compound /**  **Time (hrs)** | **1** | **2** | **4** | **7** | **10** | **24** | **48** | **72** |
| **Pimozide** | 32.93 | 12.66 | 8.25 | 5.80 | 5.93 | 8.78 | 8.09 | 3.46 |
| **Menadione** | 33.02 | 4.65 | 3.39 | 7.39 | 6.96 | 2.36 | N/A | 3.21 |
| **Manidipine HCl** | 36.14 | 7.65 | 6.13 | 4.11 | 3.34 | 2.62 | 3.83 | 3.21 |
| **Oxethazaine** | 2.95 | 3.11 | 1.86 | 2.37 | 2.65 | 3.83 | 3.36 | 2.77 |
| **Doramectin** | 100.00 | 100.00 | 16.92 | 10.46 | 7.80 | 4.08 | 2.42 | 1.59 |
| **Clofazimine** | 21.54 | 19.40 | 20.72 | 8.89 | 5.36 | 4.17 | 3.56 | 3.48 |
| **Lomerazine HCl** | 16.08 | 6.79 | 2.11 | 1.85 | 1.79 | 1.38 | 1.72 | 1.34 |
| **Flunarizine HCl** | 17.50 | 12.30 | 11.41 | 9.24 | 4.27 | 3.19 | 3.18 | 3.16 |
| **Nicardipine HCl** | 2.67 | 4.00 | 3.86 | 3.53 | 1.73 | 1.42 | 1.85 | 3.25 |
| **Fendiline HCl** | 10.65 | 7.35 | 6.60 | 4.91 | 2.39 | 1.81 | 2.93 | 2.60 |
