# Supplementary material for: Activity Profile of an FDA-Approved Compound Library against Schistosoma mansoni
Source: PLoS Negl Trop Dis. 2015 Jul 31;9(7):e0003962. doi: 10.1371/journal.pntd.0003962 (PMC4521867; doi:10.1371/journal.pntd.0003962)
Supplement: S2 Table — Green indicates a hit, red a non-hit and grey means it was lacking from the library. (DOCX) [file pntd.0003962.s002.docx]

| **Compound** | **Panic et al.** | **Abdulla et al.** | **Neves et al.** |
| --- | --- | --- | --- |
| 10-hydroxycamtothecin |  |  |  |
| 1-Phenylbiguanide |  |  |  |
| 2,3,29-triacetoxy-24-nor-1,3,5,7-friedelatetraene |  |  |  |
| 2-hydroxy-8-methoxylepidine |  |  |  |
| 4'-methoxychalcone |  |  |  |
| 5-fluoro-5'-deoxyuridine |  |  |  |
| 6-methoxyprosogerin b diethyl ether |  |  |  |
| 7,4'-dimethoxyisoflavone |  |  |  |
| Abamectin |  |  |  |
| Acacetin diacetate |  |  |  |
| Acamprosate calcium |  |  |  |
| Acetaminophen |  |  |  |
| Acetyl isogambogic acid |  |  |  |
| Acetazolamide |  |  |  |
| Aclidinium |  |  |  |
| Acriflavinium hydrochloride |  |  |  |
| Aklavine hydrochloride |  |  |  |
| Alendronate |  |  |  |
| Alexidine hydrochloride |  |  |  |
| Alpidem |  |  |  |
| Amantadine |  |  |  |
| Ambenonium |  |  |  |
| Amifostine |  |  |  |
| Aminopterin |  |  |  |
| Amitriptyline hydrochloride |  |  |  |
| Amlodipine besylate |  |  |  |
| Amoscanate |  |  |  |
| Amphotericin B |  |  |  |
| Amsacrine |  |  |  |
| Anisomycin |  |  |  |
| Antimony potassium tartrate trihydrate |  |  |  |
| Antimycin A |  |  |  |
| Apiole |  |  |  |
| Aprindine |  |  |  |
| Arachidonic acid |  |  |  |
| Arecoline hydrobromide |  |  |  |
| Aripiprazole |  |  |  |
| Arsenic trioxide diethanolamine salt |  |  |  |
| Artemether |  |  |  |
| Artemisinin |  |  |  |
| Arterolane (OZ 277) |  |  |  |
| Artesunate |  |  |  |
| Atorvastatin |  |  |  |
| Atovaquone |  |  |  |
| Atracurium besylate |  |  |  |
| Auronafin |  |  |  |
| Avocatin b |  |  |  |
| Azathioprine |  |  |  |
| Azelastine hydrochloride |  |  |  |
| Bambuterol |  |  |  |
| Benzalkonium chloride |  |  |  |
| Benzotript |  |  |  |
| Benzoxiquine |  |  |  |
| Benzthiazide |  |  |  |
| Benzyl benzoate |  |  |  |
| Betamethasone sodium phosphate |  |  |  |
| Bithionol (bithionate sodium) |  |  |  |
| Bortezomib |  |  |  |
| Bosutinib |  |  |  |
| Brinzolamide |  |  |  |
| Bronopol |  |  |  |
| Broxaldine |  |  |  |
| Bupropion |  |  |  |
| Butoconazole |  |  |  |
| Candicidin |  |  |  |
| Carbidopa |  |  |  |
| Cefdinir |  |  |  |
| Cefpiramide |  |  |  |
| Celastrol |  |  |  |
| Cepharanthine |  |  |  |
| Cetrimonium bromide |  |  |  |
| Cetylpyridinium chloride |  |  |  |
| Chlormadinone acetate |  |  |  |
| Chlormezanone |  |  |  |
| Chloroprocaine |  |  |  |
| Chlorothiazide |  |  |  |
| Chloroxine |  |  |  |
| Chlorproguanil |  |  |  |
| Chlorpromazine hydrochloride |  |  |  |
| Chlorprothixene hydrochloride |  |  |  |
| Ciclopirox olamine |  |  |  |
| Cinnarazine |  |  |  |
| Cisplatin |  |  |  |
| Citalopram |  |  |  |
| Cladribine |  |  |  |
| Clevidipine |  |  |  |
| Clofazimine |  |  |  |
| Clofoctol |  |  |  |
| Clomiphene citrate |  |  |  |
| Clomipramine hydrochloride |  |  |  |
| Clonazepam |  |  |  |
| Clotrimazole |  |  |  |
| Colchicine |  |  |  |
| Compactin (mevastatin) |  |  |  |
| Cyclobenzaprine hydrochloride |  |  |  |
| Cycloguanil |  |  |  |
| Cyclopentolate |  |  |  |
| Cyclopiazonic acid |  |  |  |
| Cycloserine |  |  |  |
| Cyclosporin A |  |  |  |
| Cyclothiazide |  |  |  |
| Cytochalasin Q |  |  |  |
| Dactinomycin |  |  |  |
| Dasatinib |  |  |  |
| Debrisoquine |  |  |  |
| Decamethonium |  |  |  |
| Dehydro (11,12)ursolic acid lactone |  |  |  |
| Demecarium |  |  |  |
| Deserpidine |  |  |  |
| Desfuranyl-17-oxokhivorin |  |  |  |
| Diazepam |  |  |  |
| Diazoxide |  |  |  |
| Dibekacin |  |  |  |
| Dibucaine |  |  |  |
| Dichlorodiphenyldichloroethylene |  |  |  |
| Dichlorophene |  |  |  |
| Dichlorphenamide |  |  |  |
| Diethylcarbamazine |  |  |  |
| Diffractaic acid |  |  |  |
| Digitonin |  |  |  |
| Digitoxin |  |  |  |
| Dihydroartemisinin |  |  |  |
| Dihydrocelastryl diacetate |  |  |  |
| Dihydrogambogic acid |  |  |  |
| Dihydrorotenone |  |  |  |
| Dimethyl gambogate |  |  |  |
| Dimethylcaffeic acid |  |  |  |
| Dipivefrin |  |  |  |
| Disulfiram |  |  |  |
| Donepezil |  |  |  |
| Doramectin |  |  |  |
| Dorzolamide |  |  |  |
| Doxepin (hydrochloride) |  |  |  |
| Dronedarone |  |  |  |
| Droxidopa |  |  |  |
| Econazole nitrate |  |  |  |
| Edrophonium |  |  |  |
| Emapunil |  |  |  |
| Epalrestat |  |  |  |
| Epoxygedunin |  |  |  |
| Eprinomectin |  |  |  |
| Ergotamine |  |  |  |
| Erythrosine sodium |  |  |  |
| Escin |  |  |  |
| Ethinamate |  |  |  |
| Ethinyl estradiol |  |  |  |
| Famciclovir |  |  |  |
| Felodipine |  |  |  |
| Fendiline hydrochloride |  |  |  |
| Flubendazole |  |  |  |
| Flunarizine hydrochloride |  |  |  |
| Flunitrazepam |  |  |  |
| Fluorouracil |  |  |  |
| Fluoxetine (hydrochloride) |  |  |  |
| Fluphenazine hydrochloride |  |  |  |
| Flubendazole |  |  |  |
| Fluvastatin |  |  |  |
| Fumagillin |  |  |  |
| Gallopamil |  |  |  |
| Gambogic acid |  |  |  |
| Gentamicin |  |  |  |
| Gentian violet |  |  |  |
| Glucitol-4-gucopyanoside |  |  |  |
| Gramicidin |  |  |  |
| Griseofulvin |  |  |  |
| Guanethidine |  |  |  |
| Halofantrine |  |  |  |
| Hesperidin |  |  |  |
| Hexachlorophene |  |  |  |
| Hexafluronium |  |  |  |
| Hexetidine |  |  |  |
| Homidium bromide |  |  |  |
| Hycanthone |  |  |  |
| Hydrochlorothiazide |  |  |  |
| Hydroflumethiazide |  |  |  |
| iclaprim |  |  |  |
| Idebenone |  |  |  |
| Imipramine hydrochloride |  |  |  |
| Inamrinone |  |  |  |
| Iodipamide |  |  |  |
| Isoflurophate |  |  |  |
| Isometheptene |  |  |  |
| Isorotenone |  |  |  |
| Isradipine |  |  |  |
| Itraconazole hydrochloride |  |  |  |
| Ixabepilone |  |  |  |
| Lasalocid sodium |  |  |  |
| Levamisole |  |  |  |
| Lomerizine hydrochloride |  |  |  |
| Loperamide |  |  |  |
| Lovastatin |  |  |  |
| Lycorine |  |  |  |
| Malathion |  |  |  |
| Manidipine hydrochloride |  |  |  |
| Mefloquine |  |  |  |
| Menadione |  |  |  |
| Mepartricin |  |  |  |
| Metformin hydrochloride |  |  |  |
| Methazolamide |  |  |  |
| Methomyl |  |  |  |
| Methotrexate |  |  |  |
| Methyclothiazide |  |  |  |
| Methylbenzethonium chloride |  |  |  |
| Methylene blue |  |  |  |
| Methylphenidate |  |  |  |
| Metitepine mesylate |  |  |  |
| Metolachlor |  |  |  |
| Metrifonate (Trichlorfon) |  |  |  |
| Mianserin |  |  |  |
| Mibefradil |  |  |  |
| Miconazole nitrate |  |  |  |
| Mifepristone |  |  |  |
| Milnacipran |  |  |  |
| Miltefosine |  |  |  |
| Mimosine |  |  |  |
| Mizoribine |  |  |  |
| Monensin sodium |  |  |  |
| Montelukast sodium |  |  |  |
| Moxidectin |  |  |  |
| Mycophenolate mofetil |  |  |  |
| Mycophenolic acid |  |  |  |
| Narasin |  |  |  |
| Natamycin |  |  |  |
| Nefazodone |  |  |  |
| Neostigmine bromide |  |  |  |
| Nicardipine hydrochloride |  |  |  |
| Niclosamide |  |  |  |
| Nifedipine |  |  |  |
| Nifuroxazide |  |  |  |
| Nifursol |  |  |  |
| Nigericin sodium |  |  |  |
| Nilvadipine |  |  |  |
| Nimodipine |  |  |  |
| Nisoldipine |  |  |  |
| Nitrendipine |  |  |  |
| Nitrofurazone |  |  |  |
| Nizatidine |  |  |  |
| Nortriptyline hydrochloride |  |  |  |
| Octisalate |  |  |  |
| Oltipraz |  |  |  |
| Orlistat |  |  |  |
| Oxethazaine |  |  |  |
| Oxybuprocaine |  |  |  |
| Oxyquinoline sulfate |  |  |  |
| Paclitaxel (Taxol) |  |  |  |
| Pararosaniline pamoate |  |  |  |
| Pargyline hydrochloride |  |  |  |
| Paroxetine |  |  |  |
| Patulin |  |  |  |
| Pectolinarin |  |  |  |
| Peldesine |  |  |  |
| Pemetrexed |  |  |  |
| Pentolinium |  |  |  |
| Pergolide mesylate |  |  |  |
| Perhexiline maleate |  |  |  |
| Perindopril |  |  |  |
| Perphenazine |  |  |  |
| Perseitol |  |  |  |
| Phenelzine |  |  |  |
| Phenylmercuric acetate |  |  |  |
| Physostigmine salicylate |  |  |  |
| Picrotoxin |  |  |  |
| Pimozide |  |  |  |
| Piroctone olamine |  |  |  |
| Podofilox |  |  |  |
| Ponatinib |  |  |  |
| Pralatrexate |  |  |  |
| Praziquantel |  |  |  |
| Prazosin hydrochloride |  |  |  |
| Primaquine phosphate |  |  |  |
| Pristimerin |  |  |  |
| Procainamide |  |  |  |
| Procaine |  |  |  |
| Prochlorperazine edisylate |  |  |  |
| Proguanil |  |  |  |
| Promazine hydrochloride |  |  |  |
| Promethazine hydrochloride |  |  |  |
| Propylhexedrine |  |  |  |
| Proscillaridin |  |  |  |
| Protoporphyrin ix |  |  |  |
| Pyridostigmine bromide |  |  |  |
| Pyrithione zinc |  |  |  |
| Pyronaridine tetraphosphate |  |  |  |
| Pyrromycin |  |  |  |
| Pyrvinium pamoate |  |  |  |
| Quinacrine |  |  |  |
| Quinethazone |  |  |  |
| Quinine |  |  |  |
| Ramipril |  |  |  |
| Reboxetine |  |  |  |
| Reserpine |  |  |  |
| Retusin dimethyl ether |  |  |  |
| Rhodomyrtoxin B |  |  |  |
| Ribavirin |  |  |  |
| Riboflavin |  |  |  |
| Riboflavin 5-phosphate sodium |  |  |  |
| Rivastigmine |  |  |  |
| Salinomycin, sodium |  |  |  |
| Sanguinarine chloride |  |  |  |
| Securinine |  |  |  |
| Selamectin |  |  |  |
| Sertraline hydrochloride |  |  |  |
| Simvastatin |  |  |  |
| Smilagenin |  |  |  |
| Succinylcholine |  |  |  |
| Sulbentine |  |  |  |
| Sulconazole nitrate |  |  |  |
| Sulfanitran |  |  |  |
| Suloctidil |  |  |  |
| Sulpiride |  |  |  |
| Suramin |  |  |  |
| Tamoxifen citrate |  |  |  |
| Tapentadol |  |  |  |
| Paclitaxel (Taxol) |  |  |  |
| Tenylidone |  |  |  |
| Terbinafine hydrochloride |  |  |  |
| Terbutaline |  |  |  |
| Terfenadine |  |  |  |
| Teriflunomide |  |  |  |
| Tetrabenazine |  |  |  |
| Tetrandrine |  |  |  |
| Thimerosal |  |  |  |
| Thioridazine hydrochloride |  |  |  |
| Thiram |  |  |  |
| Tiagabine |  |  |  |
| Tiazofurin |  |  |  |
| Tinidazole |  |  |  |
| Tolonium chloride |  |  |  |
| Tolperisone hydrochloride |  |  |  |
| Tomatine |  |  |  |
| Toremiphene citrate |  |  |  |
| Tramadol |  |  |  |
| Trichlormethiazide |  |  |  |
| Trifluoperazine hydrochloride |  |  |  |
| Triflupromazine hydrochloride |  |  |  |
| Trimethaphan |  |  |  |
| Trimetrexate |  |  |  |
| Triptonide |  |  |  |
| Verapamil hydrochloride |  |  |  |
| Venlafaxine |  |  |  |
| Vinblastine |  |  |  |
| Vinpocetine |  |  |  |
| Vorinostat |  |  |  |
| Xylazine hydrochloride |  |  |  |
| Zopiclone |  |  |  |
